# Advanced Flexible Skin-Like Pressure and Strain Sensors for Human Health Monitoring

**DOI:** 10.3390/mi12060695

**Published:** 2021-06-14

**Authors:** Xu Liu, Yuan Wei, Yuanying Qiu

**Affiliations:** 1 School of Mechano-Electronic Engineering, Xidian University, Xi’an 710071, China; 2 School of Mechanical Engineering, Xi’an Aeronautical University, Xi’an 710077, China; 3 Institute of Flexible Electronics, Northwestern Polytechnical University, Xi’an 710072, China; iamywei@nwpu.edu.cn

**Keywords:** flexible sensors, electronic skin, human monitoring, wearable electronic, pressure and strain, structure characteristic

## Abstract

Recently, owing to their excellent flexibility and adaptability, skin-like pressure and strain sensors integrated with the human body have the potential for great prospects in healthcare. This review mainly focuses on the representative advances of the flexible pressure and strain sensors for health monitoring in recent years. The review consists of five sections. Firstly, we give a brief introduction of flexible skin-like sensors and their primary demands, and we comprehensively outline the two categories of design strategies for flexible sensors. Secondly, combining the typical sensor structures and their applications in human body monitoring, we summarize the recent development of flexible pressure sensors based on perceptual mechanism, the sensing component, elastic substrate, sensitivity and detection range. Thirdly, the main structure principles and performance characteristic parameters of noteworthy flexible strain sensors are summed up, namely the sensing mechanism, sensitive element, substrate, gauge factor, stretchability, and representative applications for human monitoring. Furthermore, the representations of flexible sensors with the favorable biocompatibility and self-driven properties are introduced. Finally, in conclusion, besides continuously researching how to enhance the flexibility and sensitivity of flexible sensors, their biocompatibility, versatility and durability should also be given sufficient attention, especially for implantable bioelectronics. In addition, the discussion emphasizes the challenges and opportunities of the above highlighted characteristics of novel flexible skin-like sensors.

## 1. Introduction

Due to the favorable flexibility and adaptability, flexible wearable electronics have exhibited enormous potential in broad prospects, and consequently, they have become one of the most attractive and rapidly growing areas of novel interdisciplinary research. As the core components of flexible electronics, the excellent flexibility and outstanding sensing performance of flexible skin-like sensors are important guarantees for these flexible wearable electronics, which have become the focus of domestic and international research. Recently, flexible sensors integrated with the human body can provide powerfully diagnostic and therapeutic capabilities. A flexible sensor worn on the throat can continuously measure and interpret the coughing and breathing rates of people with COVID-19 [1]. Actually, due to their excellent flexibility and adaptability, flexible sensors are both remarkably interesting and potentially useful in a broad range of application fields [2], such as healthcare [3,4,5,6,7,8,9], human–machine interface [10,11,12], robotics [13,14,15], sensors [16,17,18] and actuators [19], bioelectronics [20,21].

It is well known that all kinds of organisms can adapt to their living environment through their unique shapes and functions, and humankind has been trying to devise structures or devices with similar biological functions by mimicking the functions and behaviors of biological systems. The advancement of science is inseparable from the learning of nature, and many researchers have attempted to simulate the complex tactile perceptual functions of the skin surface. The human epidermis is our largest and oldest sensory organ, which possess a large number of tactile receivers, including the perception of various external stimuli such as pressure, strain, temperature, humidity, vibration, and sliding. However, the unique and various tactile perception mechanisms of the skin are not yet fully understood and even poorly comprehended.

As an important medium for connecting us to the world around us, the skin not only protects us from harm but also allows us to perceive our surroundings and the properties of the objects around us. The tactile interactions of human skin are both real and immediate feeling; nevertheless, there is an underlying complex mechanism of the simple and natural skin. In addition, traditional electronic devices cannot achieve the imitation of comprehensive mechanical properties and perceptual properties of the skin. With the rapid development of flexible electronics and bionics, electronic skin based on flexible substrate materials to mimic real skin to perceive external stimuli has become a research hotspot at home and abroad, and the use of flexible electronic devices to reshape the properties of skin may have far-reaching effects on prosthetics and medicine. Consequently, flexible electronic skin has exhibited favorable application prospects in the fields of biomedicine, human–machine interface, bionic machinery, artificial intelligence, etc. With the continuous pursuit of electronic skin [22,23,24], novel materials and structural innovations with mechanical flexibility have been devised and manufactured to imitate the unique properties of human skin, including satisfactory stretchability and mechanical durability, biodegradability [25], and the paramount ability to perceive a variety of complex stimuli in large areas. Electronic skin sensors can be used for haptic sensing in wearable consumer electronics, healthcare, and robotics. Skin-like tactile sensing enables the detection of various stimuli such as pressure, strain, temperature, vibration, and sliding, and it can be used to monitor physical activity and position, or to detect vital signs, such as blood pressure [26] and respiratory rate.

**Figure 1:**
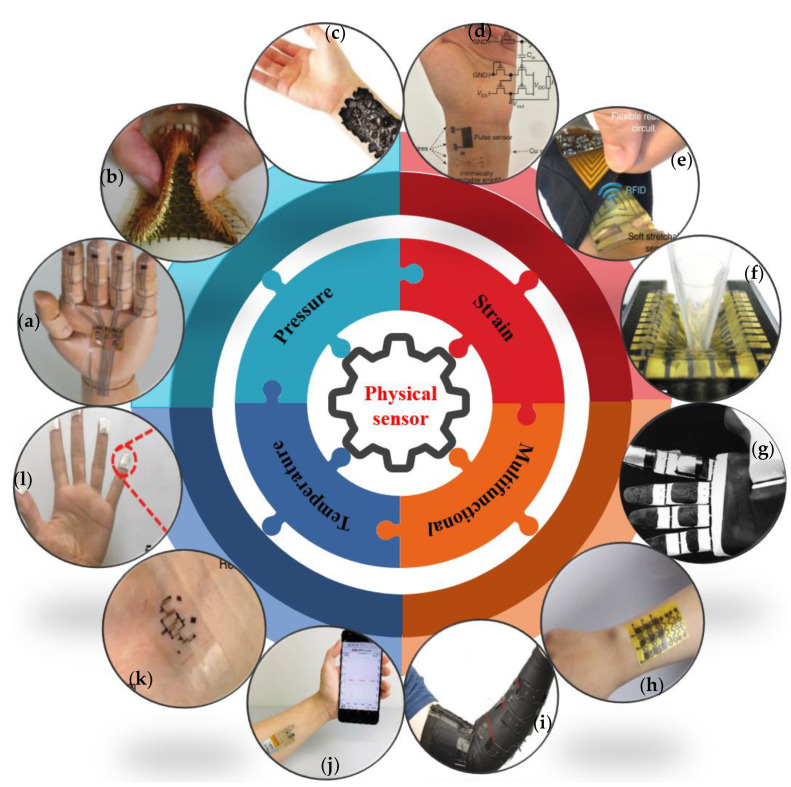
Representations of flexible physical sensors and typical applications: (**a**) A model hand with pressure sensor on the fingertips (Reprinted with permission from ref. [31]. Copyright 2015, American Association for the Advancement of Science); (**b**) Pressure–sensor integration attached to the surface of a soft balloon (Reprinted with permission from ref. [32]. Copyright 2019 Royal Society of Chemistry); (**c**) Leather-based e-skin with sensing capability by merging the sophisticated hierarchical structure of leather (Reprinted with permission from ref. [33]. 2018 The Authors); (**d**) Pulse signals measured by a stretchable strain sensor (Reprinted with permission from ref. [34]. Copyright 2018 Springer Nature); (**e**) Sensor for sensing the pulse on a human wrist (Reprinted with permission from ref. [35]. Copyright 2019, The Authors); (**f**) Skin-like stretchable strain sensor by poking with a plastic bar (Reprinted with permission from ref. [36]. Copyright 2019 The Authors); (**g**) Flexible multimodal sensor wrapped onto a finger of a robotic hand (Reprinted with permission from ref. [37]. Copyright 2016 American Association for the Advancement of Science); (**h**) The malleable e-skin can be comfortably mounted onto a human arm (Reprinted with permission from ref. [38]. Copyright The Authors); (**i**) Textile-based sensor-integrated sleeve for hand motion detection (Reprinted with permission from ref. [39]. Copyright 2020 The Authors); (**j**) Temperature sensor mounted on an arm for real-time body temperature monitoring (Reprinted with permission from ref. [40]. Copyright 2020 The Authors); (**k**) Stretchable integrated circuit for strain-independent temperature sensing (Reprinted with permission from ref. [41] Copyright 2018 The Authors); (**l**) Flexible temperature sensor attached to fingers of the hand for temperature sensing (Reprinted with permission from ref. [42]. Copyright 2019 WILEY-VCH Verlag GmbH & Co. KGaA, Weinheim, Germany).

Owing to their deformability, lightness, portability, and adaptability, flexible skin-like sensors have achieved many functions that were previously unattainable for traditional sensors. Flexible skin-like sensors can be classified into four main categories based on the quantities they measure, including physical, chemical, physiological, and multifunctional flexible skin-like sensors. Here, physical sensors are made using some physical property of the substances, which are sensitive to the measured quantities. Chemical sensors are made of sensitive elements that convert chemical quantities such as the composition and concentration of chemical substances into electrical quantities. Physiological sensors are sensors that use various biological and physiological characteristics or the properties of biological substances to detect and identify biological and physiological characteristics or chemical components in living organisms. Thereinto, pressure [27], strain, and temperature are the typical three main physical quantities that can be detected by flexible skin-like physical sensors, which can convert external physical stimulus signals such as pressure, strain, and temperature into electrical signals, thus complete the sensing function of the skin. Multifunctional sensors [28,29,30] are the integration of several different types of sensors into one unit together, which can realise the detection and testing of several variables at the same time. For example, it has been reported that the multifunctional electronic skin can achieve pressure, strain, and temperature detection simultaneously. Due to the wide range of types and applications of flexible sensors, international research on them has been very developing rapidly in recent years. Figure 1 illustrates typical applications of pressure, strain, temperature, and multifunctional flexible physical sensors.

Notably, mechanical sensing is a key functionality of flexible sensors which can be applied in health monitoring and human–machine interaction. Measuring small pressure and strain is essential to accurately evaluating external stimuli in curvilinear and dynamic surfaces. Monitoring the responses of pressure and strain can provide valuable biological information for healthcare. In recent years, numerous flexible pressure and strain sensors for human health monitoring have appeared. However, there are different types of sensors, and their performance characteristics vary greatly, and to date, there are few systematic classification summaries or comparative analyses of their applications.

Strain and pressure sensors are the most widely used for human body detection. The field is rapidly developing and flourishing with numerous articles. During the last three years, there have been numerous innovative studies in this field. Pressure and strain sensors vary greatly in terms of their mechanism, structural compositions, performance characteristics, and applications. Although there are some reviews related to stress and strain sensors, some of them mainly focus on the materials or structural preparation processes, while in other reviews, the sensing mechanisms are not systematic, and their main applications are limited. In addition, due to rapid and prosperous development, the timeliness of these reviews is poor and relatively obsolete. In general, there is a lack of systematic summary of pressure and stress sensors and their applications in human motion detection in the last three years, especially for a comprehensive evaluation of structural performance from multiple perspectives, such as sensing mechanisms, sensing elements, substrate materials, measurement ranges, and representative applications. This paper focuses on a systematic summary of the structural properties and typical applications of stress and strain sensors for human motion detection, including the four sensing mechanisms, sensitive element, substrate materials, and main performance index. The review criteria are that most of the selected papers are distinguished in representation, originality, and innovation. In addition, the majority of these papers are of high academic level and have been published in the last three years.

This paper provides an overview of the key requirement backgrounds of various flexible sensors for numerous applications, including healthcare, wearable electronics, robotic sensing, and human body integration. The paper mainly focuses on the most widely used physical sensors for human body detection, such as skin-like pressure and strain sensors, which are classified and compared according to sensing mechanisms, structure principles, and performance characteristics. The four main sensing mechanisms and representative sensors are introduced. In addition, we show the device structure components and application scenarios of these typical flexible sensors as well as list and contrast the sensitive elements and performance characteristics of different sensors. The device constituents and performance characteristics are also compared, such as sensitive elements, substrate materials, sensitivity and measurement range. The aim of this paper is to present some of the most representative structural features and device performances of flexible pressure and strain sensors, especially those used in wearable devices for human body detections. It is expected that the reader will accquire a basic understanding and appreciation of flexible pressure and strain sensors for human monitoring. However, not limited to the above, the review is also an important reference, especially for the sensors’ structural compositions, performance parameters, and numerous representative applications, such as medical health, bioelectronics, consumer electronics, human–machine integration and intelligent robotics.

## 2. The Demands and Strategies for Flexible Sensors

### 2.1. Demand for Flexible Sensors

Nowadays, people can effectively monitor the internal state of the human body through wearable devices such as smart watches and electronic vests. Apple and Samsung’s smart wearable devices are highly popular, and smart wearable devices from manufacturers such as Huawei, Xiaomi, and Royole corporations have also greatly enhanced the haptic experience for mobile phone users, giving consumers the pleasure that comes from the sense of touch. Strategies to reconstruct and restore the sense of touch via e-skin sensors are needed for a wide range of areas, such as consumer electronics, human perception, and robotic manipulation. One example is integrating nanowire transistor arrays into active matrices and sensitive rubber, which is based on the mechanism of capacitive change or piezoelectric detection to achieve tactile pressure similar to human skin and is integrated with organic light-emitting diodes to visualize transient pressure and enhance user interaction. However, most of the existing wearable products are based on hard semiconductor processes, which are not yet well suited to complex human surfaces, and they consequently bring on an uncomfortable wearing experience and make it difficult to obtain accurate data due to poor sensing accuracy. Existing wearable devices have poor wearing comfort in the event of human movement, slow response time, or make it challenging to achieve multiple functions. Moreover, during human–computer interaction of these wearable consumer electronics, the real and intimate haptic perception is profoundly dependent on the guarantee of skin-like sensors with good deformation capabilities. However, due to the non-stretchable nature of most traditional electronic materials, existing rigid and bulky electronics limit their sensing capabilities, especially for humankind, machines, and soft robots, whose activities are associated with large mechanical deformation and strain. In a word, a flexible e-skin sensor needs to conformably adhere to irregular-shaped bodies.

Nevertheless, traditional sensors are mostly based on metal and semiconductor materials, which are insufficiently flexible, portable, biocompatible, inconvenient, and comfortable, so it is difficult to adapt to the high requirements of sensors for the new generation of intelligent sensing devices. In the future, electronic sensors will become smaller, lighter, and more versatile. In the face of the increasing number of special signals to be measured and more complex environments, new materials and processes are being developed and new types of sensors are being devised and prepared. At the same time, it is urgently required that sensors will also be transparent, flexible, ductile, freely bendable, and even foldable, portable, and wearable.

**Figure 2:**
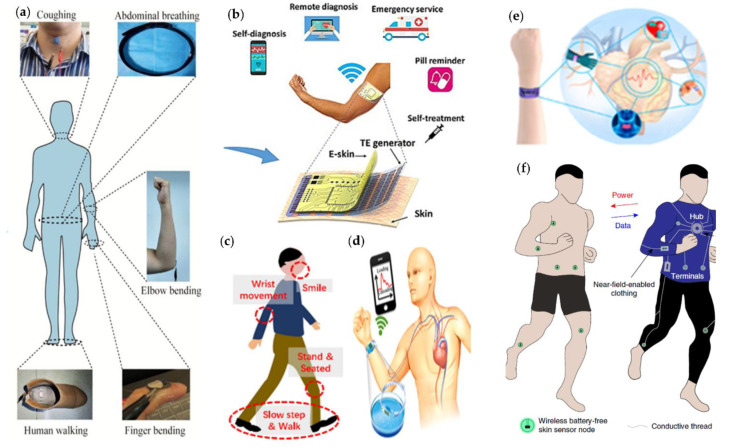
Flexible skin-like sensors for the human body. (**a**) An implantable and versatile piezoresistive sensor and application in the monitoring of human–machine interfacing (Reprinted with permission from ref. [44]. Copyright 2019 Royal Society of Chemistry); (**b**) The prospects of future e-skins powered by body heat (Reprinted with permission from ref. [45]. Copyright 2019 Elsevier); (**c**) Strain detection by SnS_2_-based sensor used for human body motion (Reprinted with permission from ref. [46]. Copyright 2021 The Authors); (**d**) Wearable transient pressure sensor for human−machine interfacing (Reprinted with permission from ref. [47]. Copyright 2019 American Chemical Society); (**e**) Flexible pressure sensor for health detection (Reprinted with permission from ref. [48]. Copyright 2020 Elsevier); (**f**) Multiple battery-free sensor nodes mounted on the skin and interconnected to a wireless reader through the near-field-enabled clothing (Reprinted with permission from ref. [49]. Copyright 2020 The Authors).

In recent years, there has been rapid development of wearable interfaces/interfaces, such as wearable electronics, electronic skin sensors [43], flexible displays, intelligent robots, and implantable medical devices. Most of these products are flexible, portable, versatile, and easy to operate due to their unique structure, and they can even be directly adhered to human skin. Figure 2 demonstrates some flexible skin-like sensors for monitoring the movements and physiological signals of the human body [44,45,46,47,48,49]. They can be integrated into our clothes or on our skin to detect health conditions and human motions without interfering with normal human movements.

In the current rapidly developing world, personal health is gradually becoming the focus of attention. Simple, fast, and convenient monitoring of human physiological conditions in real time is an important guide for stress-related health management for individuals. However, human organs have complex and variable geometric surfaces, and the human body is always in motion. The seamless integration of electronic devices into complex curved surfaces requires a high flexibility of the sensor structures. Wearable medical devices that monitor a wide range of physiological signals require flexible sensors with excellent performances. However, existing rigid and fragile conventional sensors are incapable of achieving the above functions and purposes. Hence, it is urgently and importantly necessary to obtain flexible skin-like sensors that are equipped with multiple perception capabilities and can be in close contact with various complex surfaces for long periods of time.

Moreover, with the rapid development of artificial intelligence and the internet of things, next-generation intelligent robots are also in urgent need of skin-like tactile sensors that can sense various external physical stimuli and adhere well to the complex surfaces of objects. Sensor technology is an important driver for the continuous development of robotics. For robots, body position perception or motion perception are important features for proper robot operations. Especially for soft robots, monitoring proprioception is particularly challenging because their shapes are easily deformed under external loads. With the development of robotics facing more and more complex signals and working environments, higher requirements are put forward for various sensing, and it is also hoped that sensors can also be transparent, flexible, ductile, freely bendable or even foldable, portable, wearable, etc. With the development of flexible substrate materials, flexible sensors that meet the characteristics of the above trends have emerged on this basis. Furthermore, touch sensing in human–robot interaction is critical for robots to deftly manipulate objects and safely interact with humans, such as sensing the shape, hardness, texture, and sliding of flexible sensors during robotic manipulations. These applications require sensors that are highly adaptable to complex surfaces and have favorable perception capabilities, which have stimulated research interest in electronic skin sensors. In conclusion, haptic sensing capabilities, such as pressure, strain, displacement, and temperature detection, are important for wearable medical devices, human–robot interaction, and robotic detection of the surrounding environment and object manipulation. Undoubtedly, the flexible skin-like sensor is an essential factor for realizing the next generation of multifunctional intelligent sensing electronics.

Furthermore, with the rapid development and application of graphene, carbon nanotubes (CNTs) [50], and other special materials with excellent characteristics, such as ultra-thin, tough, and having small resistivity, high-sensitivity electronic skin made of CNTs sensors can sense the tiny pressure of 20 mg such as ants on the skin. Advances in organic–inorganic hybrid electronics have enabled materials with simultaneous mechanical flexibility, hybrid ion/electron conduction, enhanced biocompatibility, and multifunctionality. Takao Someya [51] introduced an ultra-lightweight design based on a prestrained polymer elastomer for imperceptible plastic electronic that can be crumpled similarly to paper and stretched up to 230%; it could also be matrix-addressed to tactile sensors foils for health monitoring. Flexible sensors can be prepared using the inherent properties of soft polymers and organics for plastic electronic [52] due to the stretchability and mechanically adaptability, which are important qualities for interacting with biological systems, and they can be easily integrated with biological systems to form wearable and implantable devices for the interactive transfer of information between human and machine. Someya demonstrated the great potential for the brain research and treating related pathologies by novel devices for recording and stimulating neurons. Obviously, flexible skin-like sensors have become a focus of research in industry and academia due to their numerous advantages such as remarkable bendability, thinness, adaptability, and durability. To overcome the limitations of traditional rigid sensors and to improve the flexibility and adaptability of sensor structures, scientists have begun to use flexible electronic materials to prepare large-area, low-cost, and printable sensors. These skin-like sensors with flexible structures were initially called sensitive skins because of the First Symposium on Sensitive Skins, and there has been a great deal of interest in electronic skins for a variety of applications, from robotics to healthcare. V. J. Lumelsky summarized the concept and application areas of electronic-skins (E-skin) and pointed out that the E-skin is a large-area, flexible sensor array with data processing capabilities that can be used to cover the entire surface of a machine or even part of the human body, and it could also percept its surroundings through proximity, touch, pressure, temperature, chemical/biological, or other sensors. Recreating the properties of skin via electronics has profound applications in mediating our interactions with the world in a similar manner to humankind skin. Rivnay [53] described the next-generation probes, particles, and proteins for two-way communication neural interfacing for diagnosis and therapy. Obviously, it is necessary for electronic skin flexible sensors to both monitor the state of the brain and repair sensory cells.

E-skin is actually a flexible wearable bionic haptic sensor that enables smart devices to have haptic sensing capabilities via a simple primary structure, nice mechanical flexibility, and suitability for large-area fabrication. E-skin sensors are equipped with the ability to conform to a variety of complex surfaces and shapes, and they also have the ability to attach to the surfaces of smart devices similar to a straitjacket and thus help smart devices sense some basic properties of the object. However, to achieve electronic skin imitation, restore, or even replace human skin, the most important thing is to have a variety of different tactile functions at the same time; that is, human skin can sense different external pressure, temperature, humidity, and other environmental information. For example, E-skin can be used to detect the blood pressure of a sick person, breathing rate, and other physiological signals or monitor fitness, posture, gait abnormalities and other human movements. In recent years, there has been a great deal of interest in the potential of soft electronic skin-based sensors for medical and health applications. Wearable devices using flexible sensors made from soft elastic materials that are snugly attached to the human skin can detect a range of important health information, such as wrist pulse, body temperature, and blood glucose. The common force sensors, temperature sensors, physiological and biochemical sensors, and multifunctional sensors are widely used in wearable electronic devices for human health monitoring [54]. Electronic skins are used in medical and robotic applications and can help patients or robots have the ability to sense the external physical environment, besides enabling humans or robots to monitor proprioception and take appropriate action to the external environment. E-skin can mimic the various properties of human skin via the combination of new materials with excellent performance and new structural designs. Furthermore, it can also identify the geometry, spatial location, texture, and hardness of the object being touched, and it can sense strain, force, shear, twist, bending, and vibration through E-skin and thus possess sensing capability about the environment. Wang et al. [55] described the advances in skin-like sensors for robotics, human–machine interfaces, and the Internet of Things. Electronic skin sensor arrays have been made possible due to various mechanisms, innovative materials, structural designs, and advanced fabrication techniques, which have far-reaching significance for human monitoring, intelligent robotics, and human–machine interface technology.

### 2.2. Trategies for Flexible Sensors

Flexible sensor research is an important direction in the field of flexible electronics, which typically contains several key components, including the active layer, the substrate, and the interface layer. In recent years, scientists have continued to develop new materials and manufacturing strategies [56] to create skin-like wearable devices with excellent mechanical flexibility and multiple sensory functions, as well as human–machine fusion devices, which can bilaterally transmit and exchange human physiological signals. Flexible sensors can be widely used in healthcare, smart robotics, wearable devices, etc.

Currently, there are two main strategies being used to achieve structural flexibility in electronic devices: (1) inherently flexible material innovation by preparing mechanically intrinsic stretchability with favorable deformation capabilities; and (2) structural mechanics design by special mechanical structures to obtain flexibility. Even brittle or non-deformable materials have made full use of the large deformation of the structure design to absorb the stress and strain of the devices, thus avoiding damaging the material itself. Someya et al. [57] were the first to apply traditional electronic components to a robotic electronic skin, which was prepared via multiple layers of high-performance pressure-sensitive polyimide plastic and pentacene organic semiconductor. The flexible electronic skin was spined on a relatively rigid polyimide polymer into a web-like structure that achieved the ability to obtain pressure perception. John Rogers prepared submicron-scale ultra-thin strips of rigid inorganic materials such as single-crystal silicon and then bonded them to a pre-stretched rubber-like polydimethylsiloxane (PDMS) substrate. After the tension is released, the silicon deformed into waves. Thus, it ingenuously achieved structural flexibility via the organic–inorganic hybrid method. Cambridge researchers transferred stretchable circuitry to a flexible silicone substrate, giving the electronic skin similar physical properties to human skin, which can be well wrapped around the extremities and arms and is expected to be used in the field of skin transplantation in the future. The printed and transferred active materials have shown great promise for physical sensing. These flexible sensors based on printing have been applied to wearable electronics to monitor pressure, strain, temperature, and so on. Due to its variable shape and the advantages of force transfer and structural support, the traditional art of origami [58] can be used to build complex three-dimensional (3D) structures with good ductility. Moreover, the strategical combination of semiconducting and dielectric materials can reduce the fabrication complexity and enables the sensor to possess mechanical excellence and high and reliable performance. Park [59] placed MoS_2_ between Al_2_O_3_ dielectric sandwich layers to achieve the MoS_2_-based back-plane circuitry and sensor, which showed excellent linearity and response time as well as potential in sensing multi touch accurately and detecting object by grasping.

In addition, the pursuit of flexible e-skin sensors has inspired innovations in materials to imitate human skin’s unique characteristics. Microtubes can be used in novel bionic and biomedical applications such as bionic microelectronics and self-driven micro autonomous systems. Oliver G. Schmidt [15] has proposed the use of cellular robots for targeted drug delivery and reproduction by designing reconfigurable kinematic microelectronic systems. New materials and structure strategies [24] are being investigated to obtain compliant and multifunctional skin-like sensors to enable transmission signals from the body. This introduced the materials and devices designed for mimicking the skin’s ability to sense biomimetic signals. In the field of micro-nano-technology, unique 3D microstructures and nanostructures can be formed by transferring thin flexible nanomembranes, which can be used in a variety of application scenarios including flexible electronic skins and unique 3D microsystems on chips, and rolling up nanomembranes into micro-cylindrical tubes has good potential for applications in 3D electronics and photonics, sensors, and energy storages.

Moreover, numerous materials with excellent performance have now been used to prepare highly sensitive and flexible sensors due to their excellent mechanical and electrical properties, including ZnO, carbon black (CB), graphene oxide (GO), single-walled carbon nanotubes (SWCNTs), multi-walled carbon nanotubes (MWCNTs), metal nanowires, graphene nanostructures, and other conductive nanomaterials. Kausik Manna et al. [60] achieved good exfoliation and high yield preparation of graphene nanosheet lamellar structures by using a solvent mixture of N-methylpyrrolidinone (NMP) and water, and they successfully prepared highly sensitive and reliable flexible paper-based stress sensors using graphene ink formed from graphene nanosheets in a solvent controllable deoxidation–nitridation strategy. Moreover, metallic two-dimensional conductive nanomaterials are extensively employed in flexible sensors, which are promising applications ranging from health monitoring to human–machine interfaces. Nevertheless, limited to materials available and sensing abilities, Wei Huang’s team reported a controllable deoxidation and nitridation strategy [10] via the pyrolysis of an amine nitrogen source to synthesize oxygen-doped vanadium nitride (VNO) nanosheets with high conductivity.

Furthermore, as the mechanical support for materials and devices, flexible substrates play an important role in flexible electronics. Flexible skin-like sensors usually have flexible metal foils or non-metallic substrates, which include polydimethylsiloxane (PDMS), polyethylene terephthalate (PET), polyurethane (PU), polyimide (PI), polyethylene naphthalate (PEN), polyethylene oxide (PEO), polystyrene sulfonate (PEDOT: PSS), Ecoflex, and other materials. Various polymers were suitable to employ as flexible substrates to prepare flexible devices. In addition, MXene-based hybrid materials [61] with excellent features have great potential in next-generation pressure sensors for a variety of applications. The rapid development of graphene and other 2D material synthesis and new manufacturing methods for functional devices have overcome some conventional materials problems and facilitated the applications of emerging wearable flexible electronics and complex functional products. Someya et al. [62] introduced the chemical structures and properties of representative biopolymers, summarized the design and fabrication strategies for biocompatible conductors based on these biopolymers, and highlighted the fabrication techniques for various biocompatible conductors for flexible bioelectronics. Chen [63] discussed the advantages, mechanisms, and limitations of different types of stretchable conductors, both in terms of material and structural design, and they also analyzed the suitability of different types of stretchable conductors for the fabrication of flexible stretchable electronic devices. Ahn et al. [64] made the utmost of unique geometry and structural design to enable conventional materials and conventional devices to easily achieve flexibility. They also demonstrated that 2D semiconductor/semi-metallic materials can be used in complex mechanical assemblies to achieve flexible and wearable electronic devices, with typical applications including wearable haptic sensors and 3D structured photodetectors. Notably, biocompatible conductors based on natural biopolymers including proteins, peptides, and polysaccharides can be used for flexible sensor preparations.

## 3. Pressure Sensors

Humankind has always been pursuing the realization of sensors with the same ideal multi-signal perception capabilities of human skin. Flexible skin-like sensors are expected to be widely used in many areas such as healthcare, human–machine interaction, and robotics. However, subjected to the traditional sensor materials, structures, manufacturing processes, and other technical bottlenecks, there are certain difficulties for the mimicking of human haptics. Hence, it is urgently needed for high-resolution, highly sensitive, widely adapted flexible haptic sensors. In recent years, with the rapid emergence of smart medicine and health monitoring, skin-like electronic devices based on flexible sensors have received a great deal of attention. Flexible sensors can detect physical quantities, which can use certain physical effects to convert the physical quantity being measured into a signal that can be easily processed. The most common approach in current E-skin applications is generally to convert external mechanical stimuli into signal changes such as capacitance, resistance, voltage, and optics. These sensors can transform non-electric changes into electrical changes, and they can be divided into pressure sensors, strain sensors, temperature sensors, sound sensors, and acceleration sensors depending on the non-electricity of the conversion. Notably, measuring small pressure and strain are key functionalities of flexible sensors application in health monitoring. Strain and pressure sensors can provide valuable biological information regarding health conditions. Flexible pressure sensors can be used for non-invasive, high-fidelity, continuous radial artery pulse wave monitoring, which is of great significance for mobile health monitoring and remote diagnosis in cardiovascular medicine.

### 3.1. Sensing Mechanism

**Figure 3:**
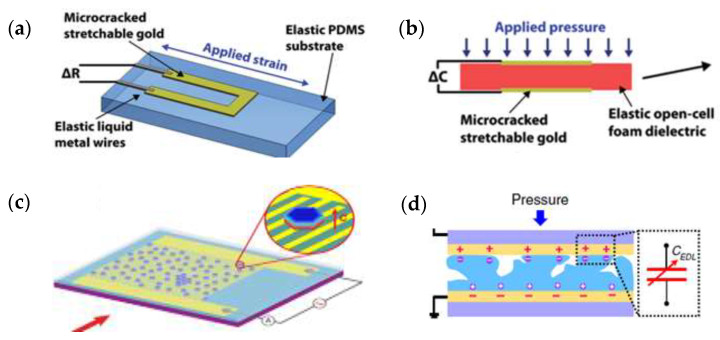
Four typical pressure sensing mechanism. (**a**) Piezoresistive (Reprinted with permission from ref. [65]. Copyright 2015 John Wiley and Sons); (**b**) Capacitive (Reprinted with permission from ref. [65]. Copyright 2015 John Wiley and Sons); (**c**) Piezoelectric (Reprinted with permission from ref. [66]. Copyright 2018 American Chemical Society); (**d**) Ionization (Reprinted with permission from ref. [67]. Copyright 2020, The Authors).

**Figure 4:**
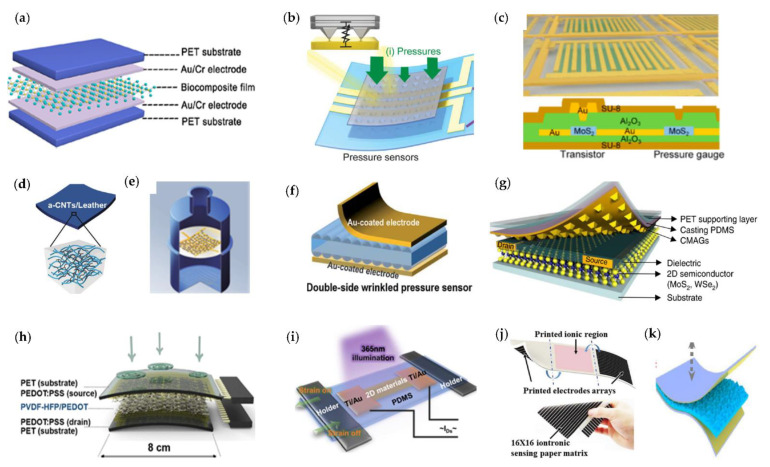
Several typical pressure sensor structures. (**a**) Schematic illustration of biocomposite film-based flexible piezoresistive pressure sensor (Reprinted with permission from ref. [48]. Copyright 2020 Elsevier); (**b**) Piezoresistive pressure sensor composed of a conducting pyramid structure (Reprinted with permission from ref. [68]. Copyright 2018 The American Association for the Advancement of Science); (**c**) Piezoresistive pressure sensor with sandwich layers (Reprinted with permission from ref. [59]. Copyright 2019 American Chemical Society); (**d**) Conductive leather with CNT for piezoresistive pressure sensor (Reprinted with permission from ref. [33]. 2018 The Authors); (**e**) Strain-based ultra-sensitive piezoresistive pressure sensor using closely spaced nanoparticle films (Reprinted with permission from ref. [69]. Copyright 2019 The Authors); (**f**) Schematic illustration of the wrinkled capacitive pressure sensors (Reprinted with permission from ref. [70]. Copyright 2017 Royal Society of Chemistry); (**g**) Capacitive pressure detection integrating conductive microstructure air-gap gate with two-dimensional semiconductor transistor (Reprinted with permission from ref. [71]. Copyright 2020 Springer Nature); (**h**) Flexible piezoelectric pressure sensors composed of PVDF/PEDOT (Reprinted with permission from ref. [72]. Copyright 2018 The Authors); (**i**) Piezoelectric senor based on Van der Waals materials (Reprinted with permission from ref. [46]. Copyright 2021 The Authors); (**j**) Flexible iontronic sensor combining traditional paper with ion sensitive materials (Reprinted with permission from ref. [73]. Copyright 2019 John Wiley and Sons); (**k**) GIA-based iontronic pressure sensor with graded intrafillable architecture between the dielectric layers (Reprinted with permission from ref. [67] Copyright 2020 The Authors).

In fact, as shown in Figure 3, there are four different methods of pressure sensing mechanism, including piezoresistive, capacitive [65], piezoelectric [66], and iontronic [67], which can be utilized to detect the magnitude of force, and each of them are relying on the respective physical effects. Figure 4 demonstrates several typical piezoresistive, capacitive, piezoelectric, and ionization pressure sensor structures [68,69,70,71,72,73]. As shown in Figure 4, there are several typical pressure sensor structures: (a) biocomposite film-based flexible piezoresistive pressure sensor; (b) piezoresistive pressure sensor with pyramid-structure; (c) piezoresistive pressure sensor with sandwich layers; (d) conductive leather piezoresistive pressure sensor; (e) ultra-sensitive piezoresistive pressure sensor using nanoparticle films; (f) the wrinkled capacitive pressure sensors; (g) capacitive pressure integrating microstructure semiconductor transistor; (h) flexible piezoelectric pressure sensors composed of PVDF/PEDOT; (i) piezoelectric senor based on Van der Waals materials; (j) flexible iontronic sensor combining traditional paper; and (k) GIA-based iontronic pressure sensor architecture. Table 1 summarizes the sensitive element materials, substrate, sensing mechanisms, sensitivities, and representative applications of several wearable skin-like pressure sensors [74,75,76,77,78,79,80,81,82,83,84,85,86,87,88].

Piezoresistive and capacitive sensors [89] capture changes in resistance and capacitance caused by geometric deformation due to external forces or strain. Piezoresistive sensors [90] convert external physical stimuli into changes in the conductive paths between conductive materials, and through the changes in resistance can easily be used to indirectly detect changes in pressure and strain by electrical test systems. Capacitance sensors detect pressure and strain by varying the frontal area and the parallel plate spacing. The detection principle is based on the change in capacitance due to the deformation/bending of pressure-sensitive mechanical elements, which changes the separation gap of the capacitor. Capacitive sensors [91] are highly sensitive, but their favorable performance is limited by parasitic noise originating from the environments.

Piezoelectric materials [92] can be deformed under mechanical pressure while simultaneously generating an electric charge, which is a piezoelectric characteristic that is the electric dipole moment resulting from the deformation of a non-centrosymmetric crystal structure. Piezoelectric sensors use the piezoelectric effect of piezoelectric materials to detect external stimuli, and highly sensitive, fast-responding and high-voltage piezoelectric materials are widely used in sensors that convert pressure into electrical signals. Hereinto, p-based lanthanum-doped zirconate titanates (PZT) and polyvinylidene fluoride (PVDF) are now very important piezoelectric materials in flexible sensor and other electronics. Wang [19] designed a highly sensitive piezoelectric acoustic sensor (PMAS) using this ultra-thin film for bionic band control, the piezoelectric voltage increased from 11 mV to 0.43 V at pressure between 0.03 and 20.4 Pa. The linear behavior of PMAS exhibits the resonant capability in the wide acoustic pressure range for microphone. Finally, based on machine learning algorithms, PMAS was carried out using biometric testing and training to reduce the error rate. Future human–computer interaction can be achieved by voice, of which flexible resonant acoustic sensors as an important component have attracted widespread attention, but the limitations of controlling multiple frequency bands and broadening the resonance spectrum do not allow comprehensive coverage of speech frequencies. The resonant bandwidth of the piezoelectric film can be broadened by employing a PZT film on an ultra-thin polymer to cover the entire speech.

Furthermore, the iontronic sensors are based on the ion-electric induction mechanism: Ionization sensing [87] use ion-selective electrodes to convert the amount of ions sensed into a usable output signal. The iontronic sensors [93] use the double-layer structure of the supercapacitor platform to create a double layer of charge on its surface; then, the interaction of the embedded electro-gel with the electrodes leads to the capacitance change of electrode as a result of squeezing the electrogel.

### 3.2. Piezoresistive Sensor

**Figure 5:**
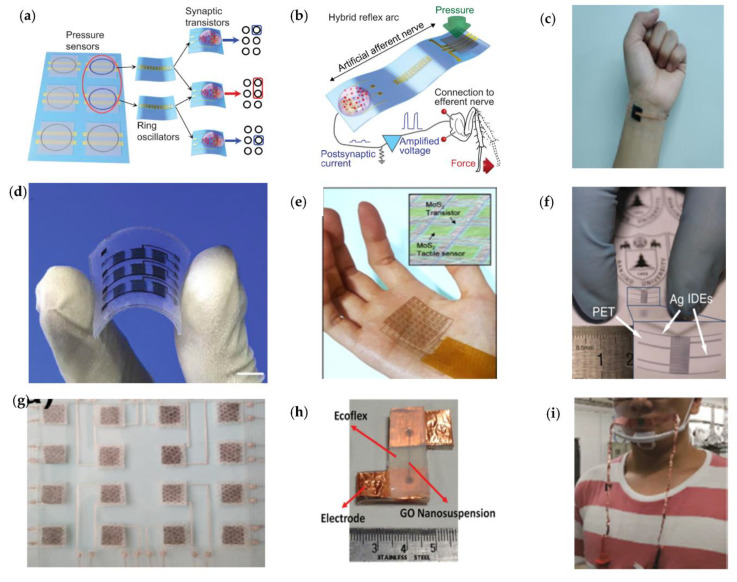
Several representative piezoresistive sensors and their applications. (**a**) Pressure sensor for artificial afferent nerve (Reprinted with permission from ref. [68] Copyright 2018 The American Association for the Advancement of Science; (**b**) Hybrid reflex arc made of an artificial afferent nerve and a biological efferent nerve (Reprinted with permission from ref. [68]. Copyright 2018 The American Association for the Advancement of Science); (**c**) The sensor attached to the wrist to monitor the pulse (Reprinted with permission from ref. [82]. Copyright 2019 American Chemical Society); (**d**) Flexible piezoresistive pressure sensor array for static and dynamic pressure mapping (Reprinted with permission from ref. [77]. Copyright 2018 John Wiley and Sons); (**e**) MoS_2_-based back-plane circuitry and sensor for detecting objects by grasping (Reprinted with permission from ref. [59]. Copyright 2019 American Chemical Society); (**f**) The pressure sensor consisting of the actuation layer and PET membrane (Reprinted with permission from ref. [69]. Copyright 2019 The Authors); (**g**) MXene/tissue-paper-based sensors with a size of 4 pixels × 4 pixels (Reprinted with permission from ref. [47]. Copyright 2019 American Chemical Society); (**h**) GO nanosuspension liquid-based flexible microfluidic tactile sensor (Reprinted with permission from ref. [74]. Copyright 2016 John Wiley and Sons); (**i**) Flexible piezoresistive pressure sensors for breathing sensing (Reprinted with permission from ref. [80]. Copyright 2019 Elsevier).

Piezoresistive pressure sensors use the principle of the piezoresistive effect of strain on the material: when the pressure changes, the strain resistance produces a change proportional to the measured pressure, and the corresponding voltage output signal is obtained from the bridge circuit. Several representative piezoresistive sensor structures and their applications are shown in Figure 5. Notably, piezoresistive sensors are ideal in biomedical applications such as cardiovascular, intracranial, urethral, and intraocular pressure measurement.

In order to enable the electronic skin to connect with the human brain nerves, Prof. Bao’s team designed a pressure sensor [68], which was composed of a conducting pyramid-structured elastomer, CNTs, and Au electrodes. An augment in pressure increases the contact area; therefore, it decreases the resistance between the CNTs electrode and the Au electrode. Based on the flexible sensor, they constructed a hybrid bioelectronics reflex arc to actuate muscles, which is expected to be applied in the fields of intelligent robotics and neuroprosthetics. Furthermore, good flexibility and adhesion are essential for sensors to conformably adhere to the skin. Such flexible sensors can be applied to the human epidermis for long periods of time to collect daily physiological data, such as pulse monitoring [86], which can be used for real-time monitoring and medical diagnosis.

An artificial afferent nerve using flexible organic electronics to mimic the functions of a sensory nerve [68] can collect pressure information (1 to 80 kilopascals) from clusters of pressure sensors; it converts the pressure information into potentials (0 to 100 hertz) by using ring oscillators and integrates the action potentials from multiple ring oscillators with a synaptic transistor. Based on the ability of animals such as chameleons in nature to change skin color, Bao’s team designed a stretchable electrochromic active e-skin with tactile sensory control by integrating a stretchable resistive pressure sensor and an organic electrochromic device, which controls the e-skin color by varying the applied pressure and the duration of the pressure.

### 3.3. Capacitive Sensor

**Figure 6:**
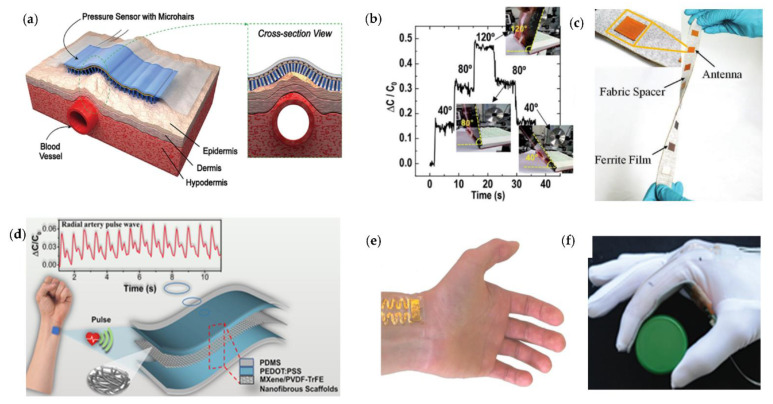
Some representative capacitive pressure sensors and applications. (**a**) Pulse-detectable pressure sensor with microhair structure (Reprinted with permission from ref. [95]. Copyright 2014 WILEY-VCH Verlag GmbH & Co. KGaA, Weinheim); (**b**) Capacitance response plot obtained from the wrinkled pressure sensor (Reprinted with permission from ref. [70]. Copyright 2017 Royal Society of Chemistry); (**c**) Textile-based flexible wireless pressure sensor(Reprinted with permission from ref. [12]. Copyright 2019 John Wiley and Sons); (**d**) Wearable capacitive pressure sensor based on MXene for reliable human physiological signals (Reprinted with permission from ref. [4]. Copyright 2020 American Chemical Society); (**e**) GO flexible capacitor-based pressure sensors attached near the wrist artery (Reprinted with permission from ref. [71]. Copyright 2020 The Authors); (**f**) Textile glove with capacitive pressure sensors (Reprinted with permission from ref. [65]. Copyright 2015 John Wiley and Sons).

In addition, the human–computer interaction of consumer smart electronic devices such as mobile phones, iTouch devices, and iPads needs to be achieved through sensors; these touch screen and fingerprint sensors are currently basically capacitive sensors. The main advantage of capacitive sensors is their high sensitivity to force, which enables the detection of small static forces with low energy consumption. Flexible capacitive pressure sensors based on various inorganic/organic materials have greatly extended the field of application of flexible wearable electronics. Some representative pressure sensors based on capacitive principle and their applications are presented in Figure 6.

Nowadays, flexible pressure sensors play a vital role in realizing lightweight, portable, and comfortable wearables for healthcare monitoring and intelligent robotics. Hence, the urgent demand for high deformability and sensibility flexible pressure will undoubtedly rise worldwide. Researchers have been trying to imitate the human sense of touch through paper-thin and ultra-sensitive artificial skin. Prof. Zhenan Bao invented a tactile-like pressure capacitive sensor made of carbon nanotubes [94], and the sensor was said to be able to clearly perceive the tactile sensation caused by a fly or butterfly resting on its surface. Then, the myriad of highly sensitive sensors can be linked together to imitate a similar function of human skin; the pressure signal will be changed into an electrical signal via flexible electronic circuits and transmitted to the brain. Noteworthily, the increasing demand for wearable devices has led to the development of highly flexible pressure sensors for monitoring various physical parameters. As one of the earliest research teams in the field of electronic skin, Bao’s team has prepared a flexible capacitive pressure sensor using microstructured polydimethylsiloxane film, which enables high sensitivity and fast response, and it can also be used for tiny object sensing and human physiological signal detection. In order to obtain sensitive pressure sensing ability similar to human skin, Bao’s team invented a highly sensitive flexible plastic film material that can mimic human skin. A portable flexible pressure sensor based on the PDMS micro-hair structure [95] was developed by Bao’s team. The team’s research electronic skin can sense the weight of a butterfly and develop electronic skin that can realize self-powered electricity as well as soft and transparent electronic skin to mimic different skin tones of human beings. The sensor can be attached to complex-shaped epidermis to increase the effective attachment area, thus achieving 12 times the signal-to-noise ratio for pressure sensing and wireless transmission. The sensor maximizes signal amplification by effective contact with complex-shaped epidermis, which has obvious advantages and can be widely used in biomedicine, smart robotics, wearable devices, etc.

Flexible pressure sensors can be attached to human skin to capture various human vital signs efficiently and transform human physiological parameters into electronic signals for human health monitoring. Some researchers have used capacitive textile pressure sensors to monitor pressure distribution at soft interfaces or sewn them into gloves to detect finger movements for better control. Nie et al. [12] proposed a textile-based flexible wireless pressure sensor, which has a sensitivity of 0.19 kPa^−1^ in the pressure range of 0–20 kPa, a high quality factor (QF > 35), high interference immunity, and good signal reproducibility after up to 20,000 cycles.

Today, it is still challenging to achieve sensors with high sensitivity, low cost, excellent mechanical stability, and ultra-low detection limits simultaneously. It is noteworthy that a capacitive pressure sensor [4] that is highly sensitive and reliable for monitoring weak physiological signals of the human body was devised, which was fabricated by a sandwich structure composed of MXene (Ti_3_C_2_T_x_)/PVDF composite nanofibers, ethylene sulfonate, and polydimethylsiloxane electrodes. The sensor achieved linear sensing over a wide pressure range (0–400 kPa), and it exhibited a high sensitivity of 0.51 kPa^−1^ and a minimum detection of 1.5 Pa. It can be used for monitoring weak physiological signals such as pulse and respiration through physiological signals as well as a human–machine interface.

### 3.4. Piezoelectric Sensor

**Figure 7:**
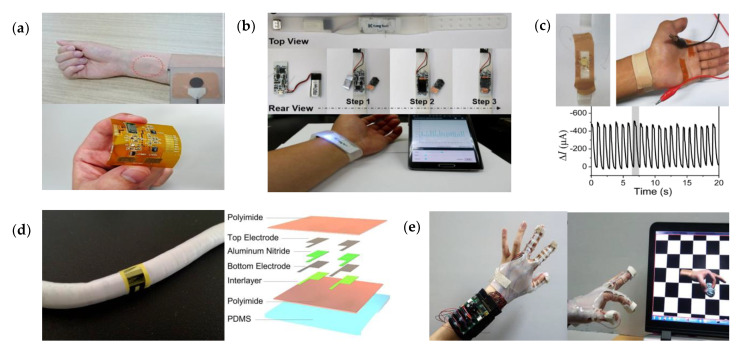
Some representative piezoelectric pressure sensors and applications. (**a**) Skin-compatible adhesive wireless sensing patch (upper) with vital sensor for pulse wave and a measuring wireless sensing system (Bottom) (Reprinted with permission from ref. [86]. Copyright 2014 Royal Society of Chemistry); (**b**) A prototype blood pressure sensor embedded in a wrist band (upper) for diagnostic and display (Bottom) (Reprinted with permission from ref. [72]. Copyright 2018 The Authors); (**c**) A flexible piezoelectric pressure sensor (upper) for the measurement of wrist pulses in 20 s real-time record (Bottom) (Reprinted with permission from ref. [85]. Copyright 2017 American Chemical Society); (**d**) A flexible smart patch sensor (left) with heterostructure (right) for the hemodynamics parameter monitoring (Reprinted with permission from ref. [96]. Copyright 2019 The Authors); (**e**) The integrated glove (left) with a sensor and actuator and actual operation (right) (Reprinted with permission from ref. [97]. Copyright 2019 The Authors).

Furthermore, piezoelectric materials can convert external pressure into a corresponding potential difference and thus can be used to measure high-frequency dynamic signals quickly and effectively. Figure 7 shows representative piezoelectric pressure sensors and applications. Among the commonly used piezoelectric materials, PVDF is now one of the most commonly used materials for flexible piezoelectric sensors due to its excellent properties such as good mechanical flexibility, good stability, large piezoelectric coefficient, and simple manufacturing process. It is reported [54] that flexible piezoelectric sensors based on the PVDF fiber array are prepared by electrospinning, and the excellent sensing capability enables the detection of tiny pressure of 0.1 Pa. However, piezoelectric sensors are very effective in detecting dynamic physical stimuli, but they usually perform poorly when measuring static signals. To address this problem of poor performance at static signals, Chen et al. [85] proposed a flexible piezoelectric pressure sensor based on a PbTiO3 nanowires (PTNWs)/graphene heterostructure. The polarized charges in the PTNWs in the sensor increased the scattering of carriers in graphene, which leaded to a decrease in carrier mobility. This heterostructured sensor has higher sensitivity than the intrinsic Chemical Vapor Deposition (CVD) grown graphene pressure sensor and is capable of measuring static mechanical signals. Such flexible piezoelectric sensors will have great potential for applications in human motion monitoring and robotics.

Human skin can sense pressure, deformation, temperature, and humidity. The flexible sensor with skin-like sensory functions can be attached to the human skin to monitor subtle changes in vital and arterial signals, which is important for detecting various health conditions such as cardiovascular disease. Young Kweon [72] proposed a facile means of fabricating flexible pressure sensors composed of poly(vinylidene fluoride-co-hexafluoropropene) (PVDF-HFP)/poly (3,4-ethylenedioxythiophene) (PEDOT). The 16 × 10 multiarray highly sensitive (13.5 kPa^−1^), large-area (8 cm × 6 cm), spatiotemporal mapping pressure sensor is based on three-dimensional electrospun conductive nanofiber, and it is available for a wireless blood pressure monitoring band. Tomohito Sekine et al. used an approximately 7.0 μC/cm^2^ polarized polyvinylidene fluoride-co-trifluoroe (PVDF-TrFE) as the pressure detection layer and designed a highly sensitive and stable flexible wearable human pulse sensor [86]. The flexible sensor with only 3 μm thickness is enough to adhere well to the skin, with a pressure sensitivity of 0.025 MPa and a response time of 0.2 s, and it can be used for continuous wireless pulse wave/rate monitoring, enabling the development of novel health monitoring medical devices.

### 3.5. Iontronic Sensors

**Figure 8:**
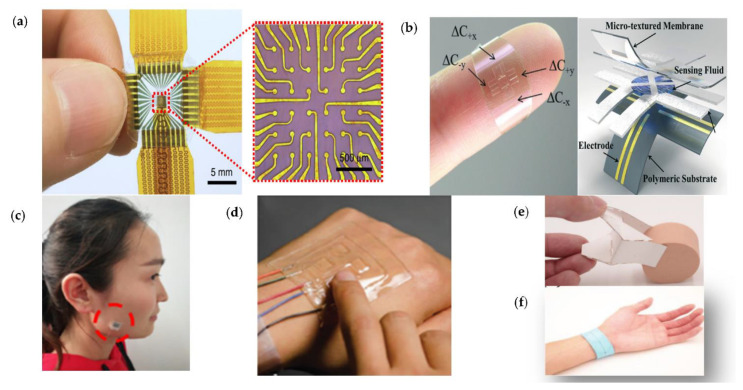
Several representative ionic sensors and their applications. (**a**) Flexible GIA-based micro-sensor arrays (left) with circular sensing pixels (Reprinted with permission from ref. [67]. Copyright 2020 The Authors); (**b**) A wearable sensor mounted on a fingertip (left) and 3D microfluidic sensing structure (right) for simultaneous tracking of the normal and shear force loads (Reprinted with permission from ref. [88]. Copyright 2014 Royal Society of Chemistry); (**c**) Pressure sensor mounted onto the cheek for real-time sensing of occlusion (Reprinted with permission from ref. [47]. Copyright 2019 American Chemical Society); (**d**) Pressure sensors (ionic skin) attached to the back of the hand (Reprinted with permission from ref. [100]. Copyright 2018 Macmillan Publishers Limited, part of Springer Nature); (**e**) Handwriting iontronic pressure sensing origami (Reprinted with permission from ref. [58]. Copyright 2019 American Chemical Society); (**f**) Wearable origami bracelet for pulse monitoring (Reprinted with permission from ref. [73]. Copyright 2019 WILEY-VCH Verlag GmbH & Co. KGaA, Weinheim, Germany).

However, existing resistive and capacitive sensors are not very highly sensitive and linear, and they have poor properties related to time response, anti-noise, and electrostatic. Several representative ionic sensors and their applications are introduced in Figure 8. Due to the development of semiconductor integration technology, based on the ion-electric induction mechanism, iontronic sensors [98] are also moving toward diversification and intelligent telemetry, and they have great expectations in the application of clinical examination and vivo embedded. These ionization sensors utilize the double layer of a supercapacitor platform in which an embedded electrogel interacts with the electrodes to form a double layer of charge on the surface whose capacitance magnitude changes due to the electrode squeezing the electrogel. Compared to the sensing mechanisms described above, ionic conductor sensors [99] via a hybrid circuit of mobile ions and mobile electrons can transmit electrical signals of high frequency over long distance. 

Hydrogels are stretchable, transparent, ionic conductors that can transmit electrical signals; the artificial skin sensor based on hydrogel can sense pressure and deformation with many distributed sensors. Prof. Zhigang Suo [100] presented an artificial skin consisting of an elastomer sandwiched between two layers of hydrogel, which was connected to a capacitance meter via two metal wires. When the artificial skin is pressed or stretched, the elastomer shape and capacitance changes for pressure sensing, and this array of pressure sensors (ionic skin) can be attached to the back of the hand. Li et al. introduced a high-performance flexible ion pressure sensor [58] via combining an iontronic sensing mechanism, the traditional origami structures, and handwriting processes. The handwritten ion-sensing origami sensor has a high sensitivity of 1.0 nF/kPa/cm^2^, a detection limit of 5.12 Pa, fast mechanical response and reset, and highly repeatable 3D sensing capability, and it is also simple, efficient, low cost, and easy to manufacture, making it highly advantageous for personalized electronics and human–machine interface applications. A novel flexible sensor with an ionic sensing mechanism [73], combining traditional paper with ion sensitive materials, successfully assigns both ionic and conductive properties to paper fibers and forms a ductile structure using a paper folding process, and it possesses a sensitivity of up to 10 nF/kPa/cm2, a high linearity, and resolution of 6.2 Pa.

### 3.6. Sensitivity

Sensitivity is a key parameter for flexible E-skins sensors; however, existing capacitive and transistor-based pressure sensors have limitations in terms of sensitivity, response speed, stability, and power consumption. In recent years, scientists have taken advantage of the fact that the tunneling conductivity between arrays of nanoparticles is extremely sensitive to the spacing of the nanoparticles to create some ultra-sensitive sensors. Chen et al. [69] prepared a piezoresistive strain-based ultra-sensitive pressure sensor using closely spaced nanoparticle films deposited on flexible membranes, using the principle that the tunneling conductance between nanoparticle arrays is extremely sensitive to the spacing of nanoparticles, and its unique quantum transport mechanism resulted in a sensor with greatly reduced thermal noise, up to 0.5 Pa resolution and up to 0.13 kPa^−1^ sensitivity. The excellent responsiveness of the sensor allows it to be used in barometric altimeters with a resolution of up to 1 m, and it has important applications in various applications, such as consumer electronics and robotics.

Furthermore, research has shown that the sensitivity of flexible E-skin sensors can be effectively improved by introducing microstructures such as miniature pyramids. Bai et al. [67] introduced a design based on the principles of graded intrafillable architecture (GIA) for the sensing mechanism, and they used graded fillable microstructures to effectively improve the compressibility and pressure response range of the structure. The microstructures can significantly improve sensitivity and extend the pressure response range, and they exhibit high sensitivity in excess of 220 kPa^−1^ over a wide range (0.08 Pa–360 kPa), with 18 Pa or 0.0056% ultra-high-pressure resolution and excellent mechanical stability. A high-sensitivity flexible sensor for the fast detection of small pressure changes is of great value in medical monitoring and robotics devices. Huang et al. [71] achieved highly sensitive, fast-response, stable, and low-power pressure sensing by integrating a conductive microstructure air-gap gate with a two-dimensional semiconductor transistor for static pressure detection, pulse measurement, acoustic wave detection, and remote pressure monitoring. The sensor has adjustable sensitivity and pressure sensing range, with an average sensitivity of 44 kPa^−1^ in the 0–5 kPa range and a maximum peak sensitivity of 770 kPa^−1^. In addition, by using the air-gap gate as a pressure-sensitive gate for the semiconductor transistor, the pressure sensitivity of the device can be amplified to 10^3^–10^7^ kPa^−1^ at 1.5 kPa.

## 4. Strain Sensor

Strain sensor [101,102] is a kind of sensor based on measuring the strain generated by the deformation of an object, which has high resolution and can measure very small strains such as 1–2 micro-strain with low error, small size, and light weight, and they can be used to measure force, moment, pressure, acceleration, etc. There is an emergent demand for high flexibility and sensitivity capable of sensing small deformations in rather broad fields, including wearable healthcare, soft robots, human–machine interaction, and so on. Table 2 summarizes the main characteristic parameters of strain sensors based on the sensing element, elastic substrate, mechanism, sensitivity, stretchability, and applications. An extremely elastic wearable strain sensor via aligned carbon nanotube fibers can be used for monitoring human motion. Sang-Gook Kim [103] designed a highly elastic stress sensor based on carbon nanotube fibers grown on a flexible substrate (Ecoflex), which induced a constant decrease in the conductive pathways and contact areas between nanotubes depending on the stretching distance. The sensor can stretch over 900% while maintaining high sensitivity, responsiveness, and durability. The biaxially oriented CNT fibers enables multi-axis strain detection and have great potential for some applications, such as strain gauge as well as single and multiaxial detection of human motion.

### 4.1. Sensor with Conventional Polymer Substrate

**Figure 9:**
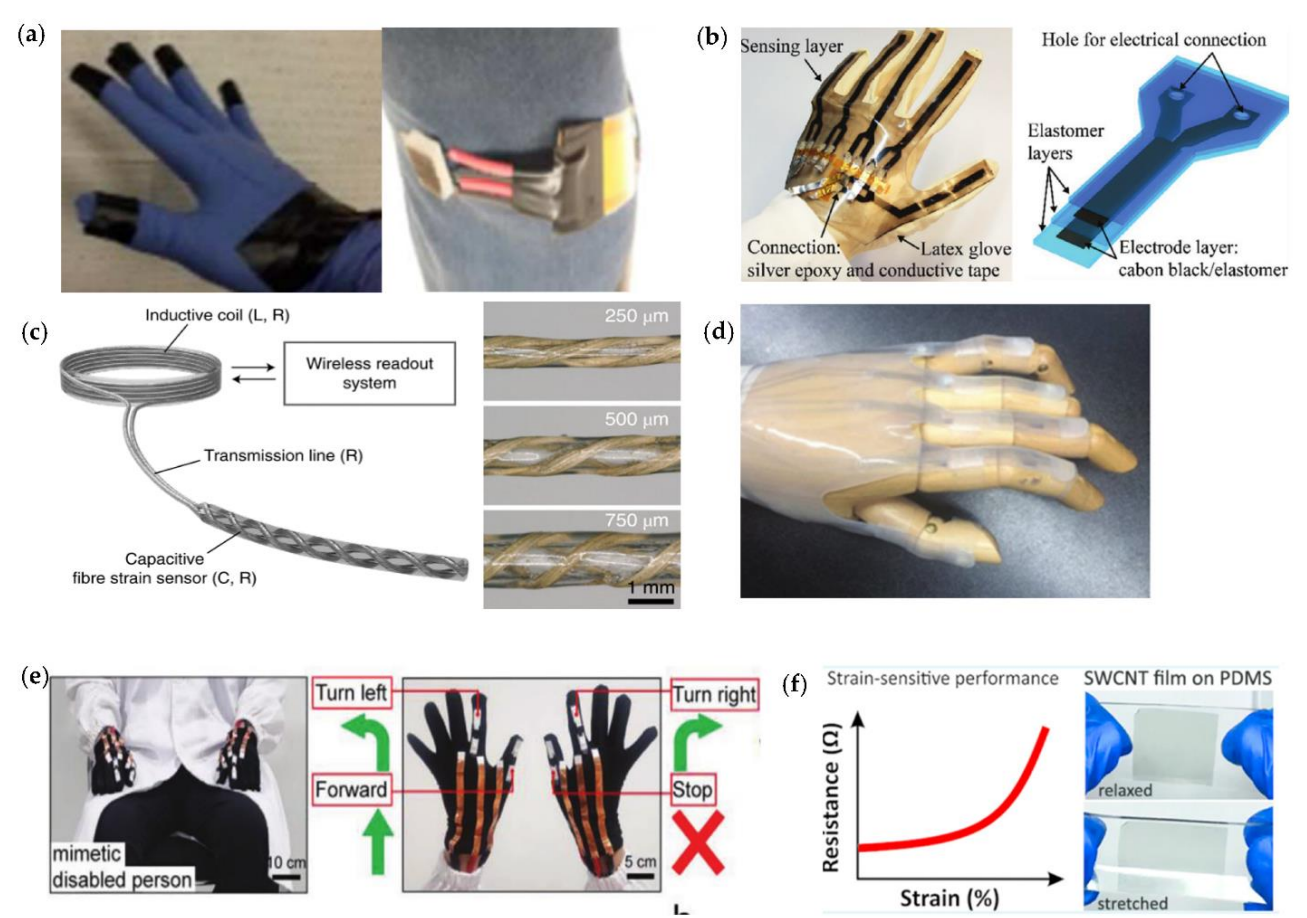
Representative of flexible strain sensors with conventional polymer substrate. (**a**) CNT-fibers-based strain sensor for monitoring human motion (Reprinted with permission from ref. [103]. Copyright 2015 American Chemical Society); (**b**) An intelligent glove (left) integrating five independently operated sensors, the sensor structure (right) consists of electrode layers and dielectric layers (Reprinted with permission from ref. [91]. Copyright 2017 WILEY-VCH Verlag GmbH & Co. KGaA, Weinheim, Germany); (**c**) A fiber strain sensor for wireless strain-sensing system (left), fiber strain sensors with various hollow-core diameters (right) (Reprinted with permission from ref. [102]. Copyright 2021 The Authors); (**d**) A glove soft sensor on a hand mock-up (Reprinted with permission from ref. [107]. Copyright 2018 Mary Ann Liebert Inc); (**e**) Smart gloves with the strain sensors attached on the finger for wireless human–machine manipulation (Reprinted with permission from ref. [10]. Copyright 2021 WILEY-VCH GmbH); (**f**) Mechanically tunable strain sensor based on SWCNT/PDMS (Reprinted with permission from ref. [104]. Copyright 2019 American Chemical Society).

Nowadays, the increasing demand for monitoring various physical parameters has led to the development of flexible strain sensors, which is one of essential factors for flexible electronics. Figure 9 shows the structures and applications of flexible strain sensors based on some common polymer substrates, including (a) a CNT-fibers-based strain sensor for monitoring human motion; (b) an intelligent glove integrating five independently operated sensors, in which the sensor structure consists of electrode layers and dielectric layers; (c) a fiber strain sensor for a wireless strain-sensing system, and fiber strain sensors with various hollow-core diameters; (d) a glove soft sensor on a hand mock-up; (e) smart gloves with the strain sensors attached on the finger for wireless human–machine manipulation; and (f) mechanically tunable strain sensors based on SWCNT/PDMS.

In order to achieve good ductility and adaptability adhered to a complex surface, flexible polymer substrates as well as ductile metal substrates are used for substrates of flexible sensors, such as common PDMS [108], PET, TPU [106], PEDOT:PSS, ECOFLEX, and other materials. Due to the advantages including easy availability, chemical stability, good transparency and thermal stability, low Young’s modulus, close to the skin to the touch, and good bonding to electronic materials, PDMS is now the preferred choice. Highly flexible and transparent strain sensors are fabricated on PDMS substrates [109], which have a favorable high sensing range of 400%, and a low creep of 4% at 400% strain. Implantable sensors can be used to continuously monitor strain information in living organisms. However, before they can be used in clinical practice, structural mismatches between weaves or organs and practical suture attachment processes, as well as wireless readout capabilities, need to be addressed. Lee at al. presented a wireless and stitchable fiber strain sensing system [102] that has been created by combining a capacitive fiber strain sensor with an inductive coil to enable wireless readout. The sensor consists of two stretchable conductive fibers in a double helix configuration with a hollow core and has a sensitivity of approximate 12, allowing effective measurement of strain in tendons and knee ligaments based on their capacitance and validation in vivo and in vitro in pack pigs.

### 4.2. Sensor with Biological Substrate

**Figure 10:**
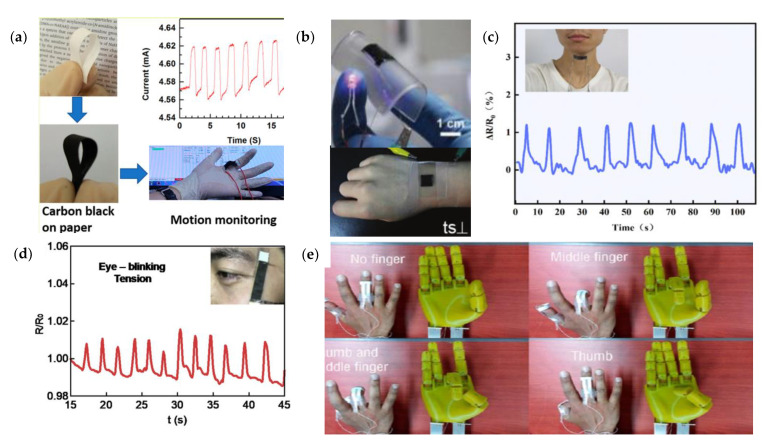
Representative of flexible strain sensors with biological substrate. (**a**) Flexible and degradable paper-based strain sensor (Reprinted with permission from ref. [110]. Copyright 2017 American Chemical Society; (**b**) Flexible and anisotropic strain sensor based on carbonized crepe paper for monitoring human wrist bending (Reprinted with permission from ref. (Reprinted with permission from ref. [112]. Copyright 2018 WILEY-VCH Verlag GmbH & Co. KGaA, Weinheim, Germany); (**c**) Strain sensor was applied to monitor drinking water (Reprinted with permission from ref. [113]. Copyright 2019 American Chemical Society); (**d**) Paper sensors for detecting eye blinking motions (Reprinted with permission from ref. [114]. Copyright 2019 Elsevier; (**e**) Foldable paper sensors based on reduced graphene oxide for control of a robotic hand (Reprinted with permission from ref. [111]. Copyright 2017 American Chemical Society).

Conventional substrates such as PET and PDMS are widely used in flexible sensors. However, the abundant and sustainable commercially available crepe paper substrate is a different substrate from above, and it is also facile, low-cost, and scalable. Figure 10 shows the structure and application of flexible strain sensors on biological substrates.

Saha [111] proposed a paper substrate wearable sensor, which was fabricated from reduced GO via desktop digital craft cutters for the masking layer. The sensor was highly sensitive to various deformations and capable of measuring as small as 0.1°, and it exhibited the applicability of detecting pulse and the motion of fingers, which were used to control a robotic hand. Furthermore, the preparation of low-cost, highly flexible electronic devices from degradable materials remains a challenge. Liu [110] designed a flexible biodegradable strain sensor by dip-coating in an aqueous suspension of carbon black (CB) and carboxymethyl cellulose (CMC). Then, the sensor was obtained by assembling the paper coated with silver paste and the wire. The relative resistance changed as a function of the tension strain in 0–0.6%. The sensor had a gauge factor of 4.3 and a response time of approximately 240 ms, it can be used to monitor various human movements, and it can be degraded soon under gentle rubbing in wet state. Inheriting from the crepe paper’s unique anisotropic structure, a high flexibility strain sensor [112] based on carbonized crepe paper can be converted into a conductive network by carbonization, the sensors perpendicular and parallel to the fibers direction exhibited gauge factors of 10.10 and 0.14, respectively.

However, the practical applications of paper-based strain sensors are still challenging due to the paper swelling and degrading after absorbing water. Recently, the problem was successively settled by an ultra-sensitive strain sensor with a superhydrophobic conductive paper substrate [113], which was fabricated by simply dip-coating printed paper into a suspension of carbon black (CB), carbon nanotubes (CNT), and a hydrophobic fumed silica (Hf-SiO_2_). The micro-crack structures in the conductive CB/CNT layer enabled the sensor to detect ultra-low strains down to 0.1%, and exhibited a sensitivity of 7.5 in the strain range of 0–0.7%. A paper-based strain sensor [114] was fabricated by molybdenum carbide–graphene (MCG) composites with porous and stacking micro-structures, which can detect both the amplitude and the direction deformation, and the gauge factors for tensile and compressive strain are 73 and 43, respectively.

### 4.3. Gauge Factors

Implantable flexible strain sensors can be used to monitor the movements of organisms continuously. Lee [102] reported a saturable fiber strain-sensing system created by combining a capacitive fiber strain sensor with an inductive coil for wireless readout. The sensor is composed of two stretchable conductive fibers organized in a double helical structure with an empty core, and the sensitivity is 12. Meanwhile, most of the gauge factors of strain sensors remain typically below 300. There is an emergent demand for high flexibility, high sensitivity, and low-power strain gauges that are capable of sensing small deformations in extreme conditions. The sensing abilities of metallic two-dimensional conductive nanomaterials need to be strengthened. Enhancing the gauge factor remains one of the greatest challenges for strain sensors. Wei Huang [10] introduced stretchable strain sensors via high VNO nanosheets, which had metallic characteristics and low dimensionality, together with layer-to-layer slippage, which was particularly suitable for capturing various physiological signals. The sensor is remarkable in performance, including having a maximum gauge factor of 2667, wide detection range of 0–100%, and 44 ms rapid response. However, the challenges related to enhancing the gauge factor and eliminating the structural mismatch between the sensors and the object still need to be addressed before they can be of use in practice. Yan [46] reported a strategy to enhance the gauge factor based on Van der Waals materials by tuning the carrier mobility and concentration through an interplay of piezoelectric and photoelectric effects. The gauge factor of the SnS_2_ sensor is up to 3933, and it can be tuned from 23 to 3933, which is demonstrated in detecting vibrations caused by sound and capturing body movements. Bao’s team introduced a manufacturing process to improve the high yield and uniformity of stretchable e-polymers, which is promising for the fabrication of next-generation stretchable skin. In order to meet the demands for future electronic skin applications, a skin-like sensor should be highly stretchable and self-healable. Recent notable advances have been made in the skin-inspired active-matrix transistor sensor array, which is capable of detecting strain distribution through surface deformation.

Jin Young Oh [36] designed a stretchable strain-sensitive and self-healable semiconducting film achieved by blending a polymer semiconductor, self-healable elastomer, and SEBS. The blend film is highly stretchable (>1300%) and self-healable at room temperature; besides, the gauge factor of the sensor reaches up to 5.75 × 10^5^ at 100% strain via controlling the percolation threshold of the polymer semiconductor. Measuring the change in contractile force of cardiomyocytes is crucial for assessing drug-induced cardiac toxicity However, most existing sensors are severely limited in their real-time applications due to low sensitivity and time-consuming processes. A cantilevered crack sensor [105], made by depositing a SiO_2_ adhesion layer on Pt and chemically bondable to a thin layer of soft PDMS, has been designed for the measurement of cardiac contraction force. The sensor has a high sensitivity of 9 × 10^6^ at 1% strain, enabling continuous measurement of myocardial contractility over a period of up to 26 days (>5 million heartbeats) while maintaining sensitivity, and the significantly flexible substrate significantly improves the reliability of the sensor compared to the absence of a PDMS layer, while allowing monitoring of drug-induced changes in contractility.

## 5. Biocompatibility and Self-Driven Capability

### 5.1. Biocompatibility

Nowadays, flexible electronics have considerably stimulated the ever-increasing demand for high performance and biocompatibility flexible sensors, which play a crucial role in the monitoring of diverse physiological signals for wearable electronics. Real-time monitoring of mechanical forces and strains on human tissues after surgical repair is required in order to develop individualized rehabilitation programs; however, existing sensors have been constrained by limitations on biocompatibility [115] and structural flexibility to meet the requirements. Nevertheless, Boutry reported an implantable pressure sensor made entirely from biodegradable elastomers [29] that degraded at the end of its useful life, eliminating secondary surgical injury. The excellent biocompatibility and detection capabilities demonstrated in rat tests indicates the great potential of the device for real-time monitoring of human tissue healing. Highly sensitive flexible and biocompatible pressure sensors have attracted significant research attention in the fields of wearable E-skin electronics [116]. Notably, biocompatible and thin organic electronic materials are considered to be ideal carriers for electronic skins. Much research attention has focused on biocompatible conductors, which are important components for soft flexible sensors; textile-based pressure sensors have been proposed for a variety of applications.

In recent years, textile-based, ultra-soft, wear-resistant, and easily integrated pressure sensors [117] have been widely used for wearable health monitoring, diagnostics, and human motion detection. Taking full advantage of the properties of fabric, Nie et al. [12] proposed a textile-based flexible wireless pressure sensor array, of which each sensing unit had a layer of soft fabric spacer sandwiched between an antenna and a ferrite film, and it enabled integration with wristbands, belts, and smart wireless insoles and application. The sensor is conducive to wearable medical and motion detection, and it can be used for remote real-time measurement of human–computer interaction pressure distribution for various soft interfaces in the future. Furthermore, compared with other polymers, natural biopolymers [118] have unique features including favorable biocompatibility, biodegradability [119], sustainability, and they are promising in various functional material properties. Recently, smart fabrics with sensing functions are the result of further integration of the traditional textile industry with sensor technology, Internet of Things technology, and other emerging technologies. Smart sensing fabrics offer potential applications as self-powered active motion sensors due to their breathability, high sensitivity, fast response time, and stable output performance, as well as simple large-scale integration that can sensitively and actively monitor human motion. Wei Huang et al. [33] combined a natural leather multi-level structure with nanomaterials to design a wearable and comfortable electronic skin with pressure-sensing capability, which can be used for the continuous monitoring of the human wrist pulse, and it can also be used for the integration of functional devices such as a back electrode display of display devices and visualization of information when the user interacts with the device. The e-skin preparation method is simple and versatile, can be combined with traditional leather processes, is conducive to low-cost scale production, and has good potential for future use in medical health and intelligent robotics. Wang [48] demonstrated a flexible pressure sensor with a 3D cross-link structure that was composed of silk fibroin@Ti_3_C_2_T_x_ MXene (SF@MXene) biocomposite films by natural silk fibroin (SF) and an MXene nanosheets wave-shaped lamellar macrostructure. The sensor with good elastic and biocompatibility exhibited a desirable sensitivity (25.5 kPa^−1^), fast response, and subtle pressure detection of limit (9.8 Pa).

### 5.2. Self-Driven Capability

With the rapid development of the Internet of Things and smart devices, the problems of traditional sensor power supply methods are becoming more and more prominent; therefore, self-powered sensors [120,121] have a very wide range of application prospects, and they are currently a hot spot for research at home and abroad. Currently, most electronic skin sensors are mainly based on external power to achieve the digital sensing of physical quantities such as pressure and position. However, Prof. Zhang [122] has developed a novel self-driven flexible and transparent multifunctional electronic skin, which used PDMS with a microstructured surface and modified fluorocarbon polymer as the friction surface, and it achieves fully self-driven sensing and 1.9 mm positioning accuracy by using the frictional charge generated by the contact between the target object and the surface of the electronic skin, and it is able to sense the pressure of a bee limb (≈0.16 g) falling on the e-skin. The team proposes a novel self-driven sensing mechanism for the energy supply [123] problem that has long bound the development of electronic skin, and they systematically summarize the progress of the design and application of new bionic electronic skin based on friction nanogenerators in recent years, which provides the possibility of energy supply for electronic skin.

Currently, most flexible sensor devices based on capacitive, piezoresistive, and optoelectronics require an external power supply, while friction nanogenerators (TENG) can be self-driven and have the advantages of flexible design, low cost, stable output performance, etc. The use of flexible sensors in friction nanogenerator yards can achieve the detection of static processes and dynamic processes. Jiang et al. designed a self-powered hybrid sensing system integrated with flexible pressure sensors and a TENG matrix [124], which enabled the rapid detection and visualization of pressure information with sensitivity up to 190 kPa^−1^ and 500 microns resolution for multi-touch sensing. The visualized flexible pressure sensors are promising for use in motion monitoring, instantaneous pressure detection, and mapping of real-time pressure distribution, and they have potential for stress/strain sensing of smart sensor networks for wireless detection and communication in human–machine interfaces. Embedded flexible sensors are the key to safe and dexterous gripping by soft robot grippers. A self-powered, flexible multifunctional sensor [13] is composed of PVDF and a microstructured jamming layer, and it is fabricated by a multimaterial 3D printing. The sensor built into the soft finger can detect the curvature and hardness of objects with a sensitivity of 0.55 × 10^−3^ V·m and 0.09 V·m/N, respectively, and the hardness of the finger can be adjusted without affecting the robot’s motion performance. The tactile sensor significantly improves the way the robot interacts with the manipulated object.

## 6. Conclusions and Outlook

In this review, firstly, we briefly introduce the concepts, key demands, and preparation strategies of flexible skin-like sensors. Secondly, flexible pressure and strain sensors for human monitoring are summarized respectively, including sensing mechanisms, sensing elements, flexible substrates, sensitivity, measurement ranges, and representative applications. In addition, pressure and strain sensors with excellent biocompatibility and self-driven capability are introduced. Finally, the article concludes with the challenges and opportunities in sensitivity, biocompatibility, versatility, and durability for flexible skin-like sensors. The majority of current research has continuously researched on how to enhance the flexibility and sensitivity of flexible pressure and strain sensors; however, biocompatibility, versatility and durability should also be given sufficient attention, especially for implantable medical devices, human–machine integration, and bioelectronics.

At present, most research of flexible pressure and strain sensors aims at improving flexibility and adaptability to make the electronic–human body interface as seamless as possible. The ultimate goals of flexible sensors are seamless and 3D curved attachment, dynamical multipoint and multimodal sensing, as well as bidirectional interfaces. In terms of flexible sensors, the ability to detect touch, stretch and bend has been achieved, but to differentiate them between multiple actions at the same time remains a challenge. The development of heterogeneous structures used in flexible electronics presents an interesting set of challenges, and further research is still urgently needed to explore how to realize all these materials integrating and working together, as well as managing interface and mechanical property mismatches between soft and hard materials. The cost-effective sensing systems have great potential in human–machine interfaces, virtual reality manipulation, robotics automation, healthcare, and other areas. However, most soft robots generally use elastomers, which possess nonlinear, hysteretic and viscoelastic properties, which consequently make monitoring proprioception more difficult. There has been a lot of research on flexible skin-like pressure and strain sensors in recent years, most of which focuses on how to improve the performance of the sensors in flexibility and sensitivity. However, different definitions of sensors’ sensitivity have been reported in the previous literature; there is no clear and unified standard of the definition of sensitivity for flexible sensors. Furthermore, the broad ranges and varying criterias of sensitivities result in difficulties for the comprehensive evaluations of their performances.

In addition, advances in the internet of things, big data, and digital healthcare will drive innovations in high performance wearable and smart sensors. Diversified sensors are the mainstream technology to realize human–machine interfaces and automation controls. Numerous studies have investigated multifunctional sensing, which can be integrated with many different measurement quantities and sensing mechanisms of multifunctional sensors. Nevertheless, multiple sensors also bring about redundant and even contradictory informations. Therefore, in order to achieve a consistent interpretation and description of the environment, the informations collected from the various sensors and their monitoring must be combined according to certain optimization criterias. The multifunctional sensors urgently require the further processing of information fusion via multilevel, multifaceted and multilayered processing of data to obtain a comprehensive and better estimate. However, there are still many areas that need to be further explored regarding high sensitivity, adaptability and reliability. As soon as these issues are resolved, the sensors can work independently and effectively in the measurement of different types of signals simultaneously without affecting each other. The majority of the literature reports on essentially contact sensors, but non-contact sensors possess more advantageous than contact sensing in cases such as COVID-19. The different types of non-contact have their own advantages; capacitive sensing is more commonly reported, but magnetic and frictional sensing are relatively rare.

Furthermore, textile-based flexible sensors also have key issues to address in biomedical and robotic fields such as wiring complexity, poor resilience, and signal fluctuations due to environmental changes. For practical applications, there is still much further work to be done. Such sensing systems can be made from bio-absorbable materials to avoid the secondary damage of removal surgery, and can achieve better biocompatibility and wearable reading systems that can continuously read response signals in real time. Flexible sensors will be exposed to a variety of prolonged stresses under various loads in practical applications, and they need to be adhered consistently to irregularly shaped surfaces during the operations. However, these flexible sensors may fail due to the fatigue, corrosion or damage of the device under a long lifetime of service. Similar to biological systems with strong self-healing capabilities, future electronic skin sensors for human skin can achieve autonomous healing capability to restore its mechanical and electrical properties. The preparations of self-healing soft electronic materials, self-healing electronic devices, and their potential new functions will become scientific problems to be solved urgently.

Finally, due to the superior mechanical properties such as bendability and stretchability, stretchable flexible skin-like sensors have broad prospects in health, medical, artificial skin, human–machine interfaces, and the internet of things. However, it remains challenging to confer these desired functionalities to an active semiconductor. Wearable electronic devices still face great challenges in terms of flexibility, light weight, and breathability. On the other hand, smart touch sensing systems are crucial for human–machine interfaces, and although artificial skin sensors have the advantages of high sensitivity and shaped adaptabilitiy, they still need significant improvements in skin-friendliness, breathability and comfortability. With the perfect combination of flexible sensor and smart watches, wristbands and other wearable devices, it is only necessary to analyze the electrical signal output from the electronic skin to realize the “intelligent pulse”. Wireless-controlled and powered skin-integrated virtual reality (VR) systems will make it possible to feel the hug of a friend or relative real when talking to them visually, which could soon be a reality. Scientists have continued to develop new human–machine fusion device and multiple sensory functions, which can bilaterally transmit and exchange human physiological signals. As one of the currently most important scientific and technological representations, brain–machine interfaces are promising to promote the distinct mechanism of how the brain works and provides new tools to diagnose and treat various diseases, but they are still somewhat limited by organic materials, which integrate with tissues and transmit signals at the biotic/non-biotic interface. The ultimate goals for future electronic skin should be to mimic or even surpass the properties of human skin, and can be integrated with electronic devices or actuators that have sensory feedback, just as human skin can quickly respond to environmental stimuli while transmitting the corresponding signals to the brain and guiding muscles to take responsive actions.

In conclusion, the recent surge of interests in flexible sensor researches are significant for promoting further explorations. Only when electronic skins truly improve the lives of humankind, will they generate strong impetus for fundamental researches with the investment of more research resources. Besides the most basic capabilities of structural flexibility and perception, multifunctional sensing, biocompatibility, self-healing and biodegradability will become more crucial to smart wearable e-skin sensors. Furthermore, with the rapid advance of flexible electronics, artificial intelligence, big data and other related technologies, flexible wearable skin-like sensors will certainly give birth to abundant intelligent wearable devices and bionic robots with powerful haptic sensing functions. These flexible skin-like sensing systems will profoundly affect the future technological world, thus human daily life will be much more intelligent and humanized with multiple sensing functions.

## Figures and Tables

**Table 1:** Summary of main characteristic parameters of pressure sensors based on sensing elements and elastic substrates.

Sensing Element	Substrate	Mechanism	Sensitivity	Detection Limitation	References
CNTs/leather	Leather	Piezoresistive	8.03 kPa^−1^–32.42 kPa^−1^	50 kPa	[33]
MXene/Ti_3_C_2_Tx	Paper	Piezoresistive	3.81 kPa^−1^	23 Pa−30 kPa	[47]
SF@MXene	PET	Piezoresistive	25.5 kPa^−1^	0.1 kPa–20 kPa	[48]
AM/MoS_2_/Al_2_O_3_	Microchem SU8	Piezoresistive	0.011 kPa^−1^	1–120 kPa	[59]
CNT and Au	Elastomer	Piezoresistive	/	1–80 kPa	[68]
Nanoparticles	PET	Piezoresistive	0.13 kPa^−1^	0.5 Pa	[69]
Graphene oxide	Ecoflex/PDMS	Piezoresistive	0.0338 kPa^−1^	7 mN	[74]
CB and NaCl	PDMS	Piezoresistive	5.54 kPa ^−1^	10 Pa–800 kPa	[75]
Graphene oxide	PDMS	Piezoresistive	25.1 kPa ^–1^	0–2.6 kPa	[76]
Graphene	PDMS	Piezoresistive	1.2 kPa ^−1^	5 Pa–25 kPa	[77]
Graphene/Ag	Sea sponge	Piezoresistive	0.016 kPa ^−1^	0–40 kPa	[78]
Carbon/MXene	PET	Piezoresistive	12.5 kPa^−1^	0–10 kPa	[79]
Ti_3_C_2_T_x_ MXenes	PU sponge	Piezoresistive	0.01 kPa^−1^	9 Pa–245.7 kPa	[80]
PEDOT:PSS	Paper	Piezoresistive	1.14 kPa^−1^	300 Kpa	[81]
CB/AP	Paper	Piezoresistive	51.23 kPa^−1^	1 Pa	[82]
MXene/PVDF	PDMS	Capacitive	0.51 kPa^−1^	0–400 kPa	[4]
Ferrite	Fabric	Capacitive	0.19 kPa^−1^	0–20 kPa	[12]
Silicone/Gold	PDMS	Capacitive	0.001–0.01 kPa^−1^	5–405 kPa	[65]
MoS_2_/WSe_2_	PET	Capacitive	44 kPa^−1^	0–5 kPa	[71]
ZnO	PDMS	Piezoelectric	84.2–104.4 meV/MPa	0–1 Mpa	[66]
PVDF-HFP/PEDOT	PET	Piezoelectric	13.5 kPa^−1^	1 Pa–30 kPa	[72]
ZnO	Cr/Au	Piezoelectric	1448–1677 meV/MPa	24.84–152.88 kPa	[83]
ZnO	Cr/Au	Piezoelectric	60.97–78.23 meV/MPa	2 kPa–3.64 MPa	[84]
PbTiO_3_/Graphene	PDMS	Piezoelectric	9.4 × 10^−3^ kPa^−1^	0–1.5 kPa	[85]
PVDF	PEN	Piezoelectric	25 kPa^−1^	0.025–1.5 MPa	[86]
Conductive fiber	Paper	Iontronic	1.0 nF/kPa/cm^2^	5.12 Pa–200 kPa	[58]
PVA/H_3_PO_4_	PI	Iontronic	220 kPa^−1^	0.08 Pa–360 kPa	[67]
Cellulose fiber	Paper	Iontronic	10 nF/kPa/cm^2^	6.25 Pa	[73]
Emim TCM	PET	Iontronic	0.43 nF/kPa/cm^2^	33 Pa	[87]
Emim TCM	PET	Iontronic	29.8 nF/kPa/cm^2^	100 mN	[88]

Abbreviations: Airlaid paper (AP); Active-matrix (AM); Silk fibroin@Ti_3_C_2_T_x_ MXene (SF@MXene); Poly(vinylidene fluoride-co-hexafluoropropene) (PVDF-HFP), Poly(3,4-ethylene-dioxythiophene) (PEDOT), (PVDF-HFP/PEDOT); Polyvinyl alcohol (PVA); 1-ethyl-3-methylimidazolium tricyanomethanide (Emim TCM).

**Table 2:** Summary of main characteristic parameters of strain sensors based on sensing element and elastic substrates.

Sensing Material	Substrate	Mechanism	Gauge Factors	Stretchability	References
SnS_2_	PDMS	Piezoelectric	23–3933	1.4%	[46]
CB	Ecoflex	Capacitive	0.83–0.98	50%–500%	[91]
Ag/PU	PDMS	Capacitive	12	0–40%	[102]
VNO	PDMS	Piezoresistive	2667	0–100%	[10]
PDMS-PDCA	SEBS	Piezoresistive	5.75 × 10^5^	100%	[36]
SWCNTs	PDMS	Piezoresistive	2	60%	[50]
GO/AgNWs	Sponge	Piezoresistive	1.5	0–60%	[78]
CNT	Ecoflex	Piezoresistive	0.54–64	900%	[103]
SWCNTs	PDMS	Piezoresistive	20.1	10%–100%	[104]
Si rubber	PDMS	Piezoresistive	166.6	0.7%	[105]
MWCNTs	TPU	Piezoresistive	1.5–3	50%	[106]
EGaIn	Silicone	Piezoresistive	2.5	0–100%	[107]
MWCNTs	PDMS	Piezoresistive	7.22	40%	[108]
SACNT	PDMS	Piezoresistive	0.1	400%	[109]
Carbon black	Paper	Piezoresistive	4.3	0.6%	[110]
GO	Paper	Piezoresistive	66.6	6%	[111]
Carbonized paper	Paper	Piezoresistive	0.14–10.1	5%	[112]
CB/CNT	Paper	Piezoresistive	7.5	0.7%	[113]
MCG	Paper	Piezoresistive	73	0.25%	[114]

Abbreviations: Poly(dimethylsiloxane-alt-2,6-pyridinedicarbozamine) (PDMS-PDCA); Polystyrene-block-poly(ethylene-ran-butylene)-block-polystyrene (SEBS); Thermoplastic polyurethane (TPU); Eutectic gallium–indium (EGaIn); Super aligned CNT (SACNT); Vanadium nitride (VNO); Molybdenum carbide-graphene (MCG); Gauge factors (GF).

## Data Availability

No new data were created or analyzed in this study. Data sharing is not applicable to this article.
